# Supplementary material for: Mechanical compression regulates tumor spheroid invasion into a 3D collagen matrix
Source: ArXiv. 2023 Jul 3:arXiv:2307.01289v1. Preprint. [Version 1] (PMC10350096)
Supplement: 1 [file NIHPP2307.01289V1-supplement-1.pdf]

Supplementary Figures:

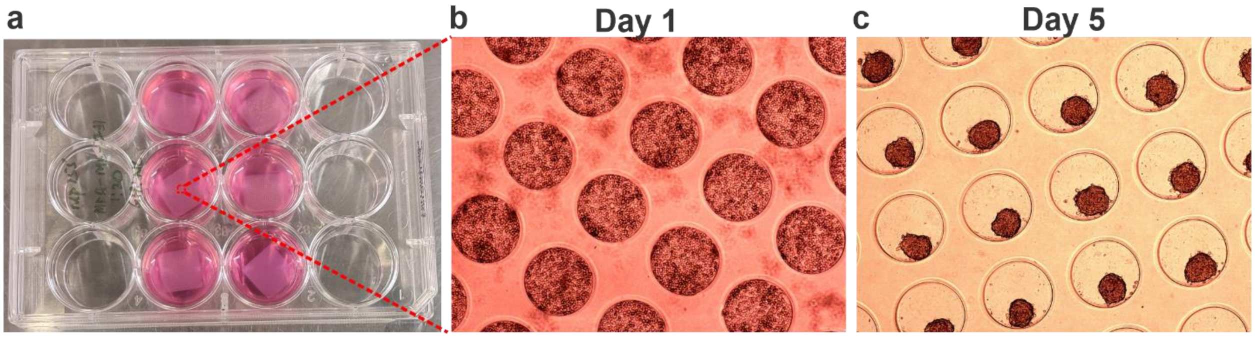

Figure S1: High throughput tumor spheroid formation assay. A) Image of a 12 well plate containing high throughput agarose based microwell array for spheroid formation. Each microwell device has 18x18 microwells patterned agarose gel membrane, and each microwell is 400 µm in diameter and depth. In a typical experiment 2 of these arrays are used to harvest spheroids. **B)** Micrograph of tumor spheroid formation in a microwell array. A total of 3 million MDA-MB-231 cells are seeded on day 1 to each microwell array. **C)** The prepared spheroids are harvested on day 5 and the media is changed regularly every 2-3 days. MCF10A spheroids were made in the same device.

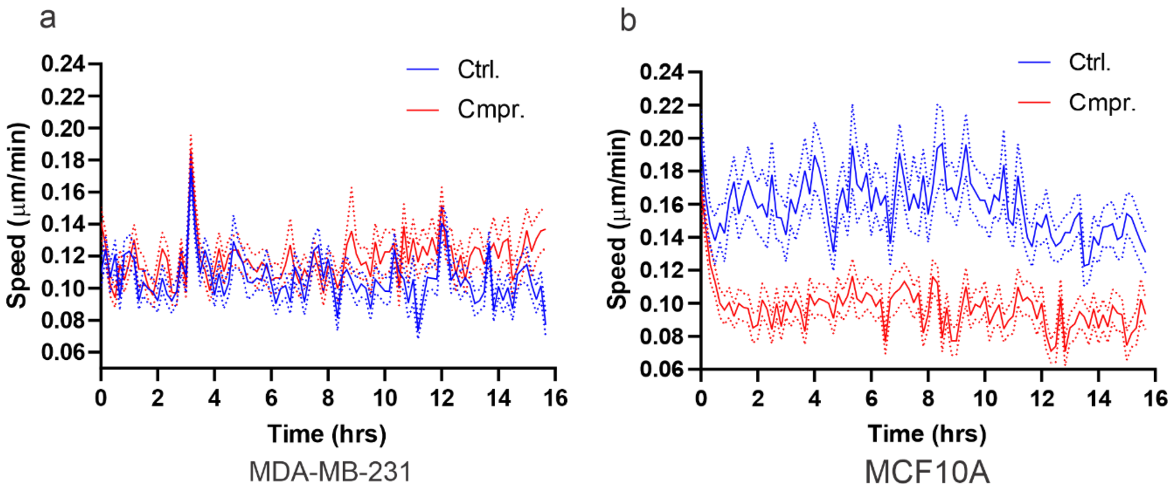

Figure S2: Time evolution of speed in MDA-MB-231 and MCF10A spheroids. A) Speed of MDA-MB-231 cells within spheroids **B)** Speed of MCF10A cells within spheroids as a function of time under compressed and control conditions.

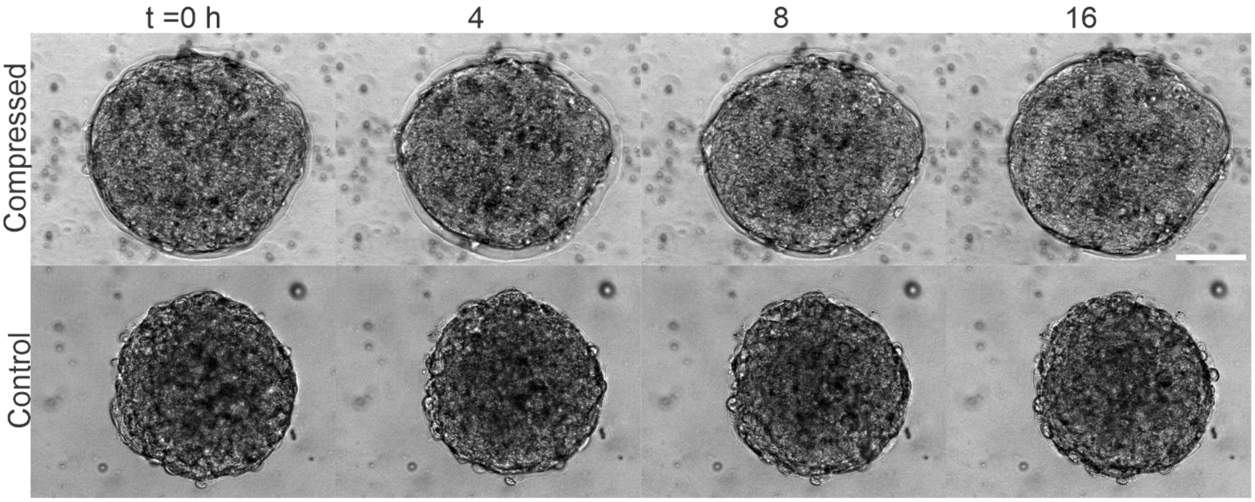

Figure S3: Compaction of MCF10A spheroids in 3.5 mg/ml collagen. Micrographs of MCF10A spheroids embedded in 3.5 mg/ml collagen taken at t = 0 hours for compressed and control condition. Scale bar is 50 µm.
